# Editorial: The role of vitamin D as an immunomodulator, volume III

**DOI:** 10.3389/fimmu.2026.1951698

**Published:** 2026-08-11

**Authors:** Mourad Aribi, Franck J. D. Mennechet, Chafia Touil-Boukoffa

**Affiliations:** 1 Laboratory of Applied Molecular Biology and Immunology, University of Tlemcen, Tlemcen, Algeria; 2 Algerian Society of Experimental and Applied Immunology (ASEAI), Tlemcen, Algeria; 3 China-Algeria International Joint Laboratory on Emergency Medicine and Immunology, Guangdong Provincial People’s Hospital (Guangdong Academy of Medical Sciences), Southern Medical University, Guangzhou, China; 4 Biotechnology Research Center (CRBt), Constantine, Algeria; 5 Institut National de la Santé et de la Recherche Médicale (INSERM) U1058, University of Montpellier, Montpellier, France; 6 Algerian Academy of Sciences and Technologies (AAST), Algiers, Algeria

**Keywords:** allergic diseases, asthma, immunomodulation, Kawasaki disease, precision nutrition, thyroid-associated ophthalmopathy, transcriptomics, vitamin D

## 1 Introduction

Vitamin D is a pleiotropic secosteroid hormone that extends far beyond its classical role in calcium and phosphate homeostasis to emerge as a pivotal regulator of innate and adaptive immunity (1–3). Following cutaneous synthesis under ultraviolet B (UVB) radiation or dietary intake (4), vitamin D is sequentially hydroxylated in the liver to 25-hydroxyvitamin D [25(OH)D] and subsequently converted into its biologically active metabolite, 1,25-dihydroxyvitamin D_3_ [1,25(OH)_2_D_3_], primarily in the kidney (5, 6) but also locally by numerous immune cells expressing the enzyme CYP27B1, including monocytes, macrophages, dendritic cells, activated T-cells, and B-cells (7–10). The active metabolite exerts its biological effects through binding to the intracellular vitamin D receptor (VDR), a ligand-activated transcription factor widely expressed in both immune and non-immune cells, which heterodimerizes with the retinoid X receptor (RXR) and regulates the expression of hundreds of target genes through vitamin D response elements (VDREs) in the genome, thereby orchestrating extensive transcriptional programs involved in antimicrobial defense, inflammation, immune tolerance, cellular differentiation, proliferation, and tissue repair (11–13). Through this integrated endocrine, paracrine, and autocrine signaling network, vitamin D orchestrates multiple aspects of host immunity by strengthening innate defense mechanisms, modulating adaptive immune responses, maintaining immune homeostasis, and preventing excessive inflammation through the regulation of antigen-presenting cells, T-cell polarization, regulatory T-cell development, B-cell function, and cytokine production (14, 15). Accumulating experimental, translational, and clinical evidence has consequently implicated vitamin D signaling in the pathophysiology of a broad spectrum of immune-mediated disorders, including autoimmune diseases, allergic and inflammatory conditions, infectious diseases, cancer, and emerging syndromes characterized by dysregulated immune responses such as sepsis and acute respiratory distress syndrome (16–20), although the magnitude and clinical relevance of these effects remain highly dependent on the underlying disease, tissue microenvironment, and individual immunometabolic context.

**Figure 1:**
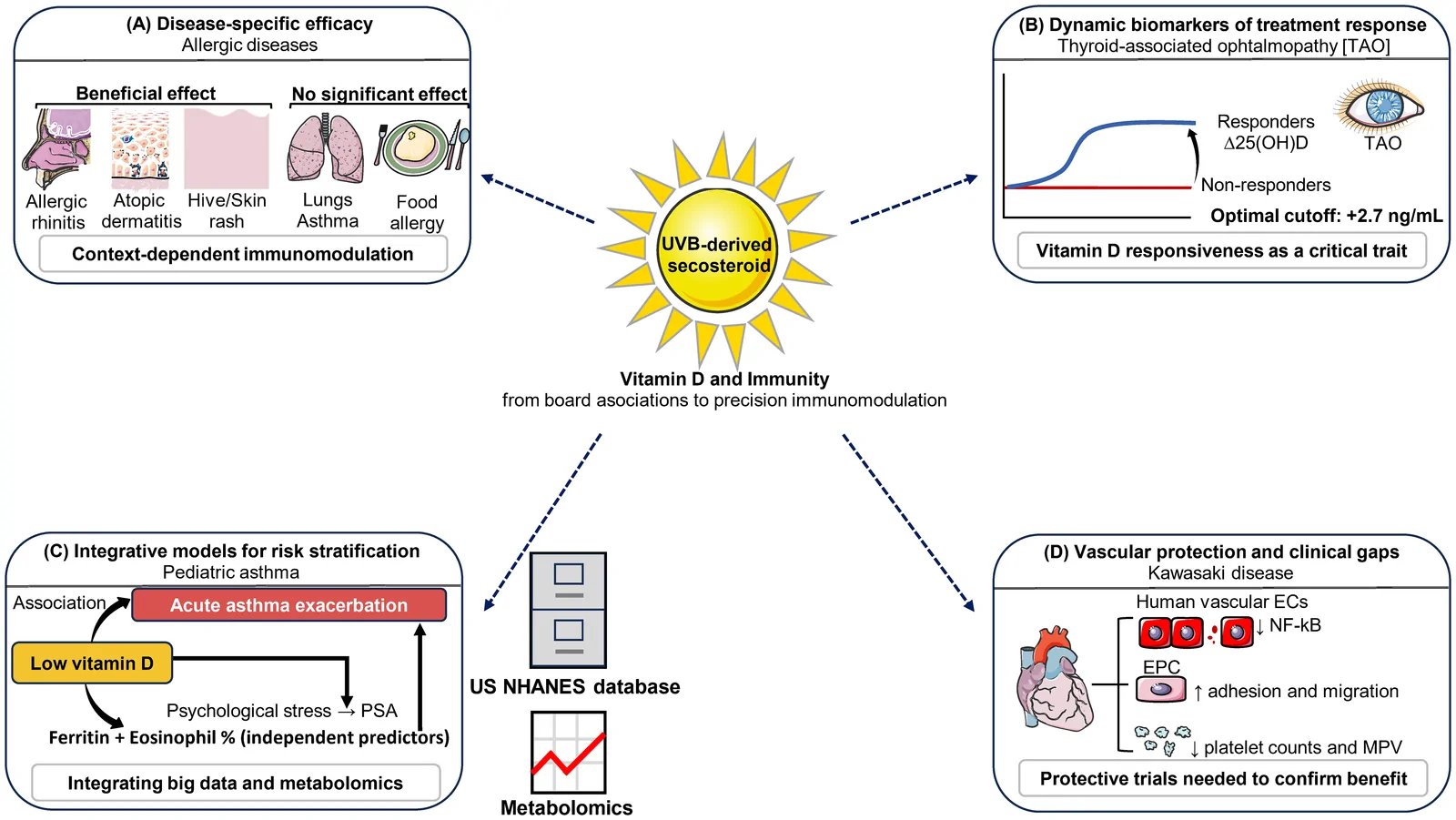
Moving towards precision immunomodulation: key insights from volume III. The central panel highlights vitamin D and immunity as the foundational hub bridging broad associations to precision immunomodulation, anchored by the UVB-derived secosteroid nature of vitamin D. Radiating outward are four key pillars: **(A)** Disease-specific effects in allergic diseases. An umbrella review demonstrates that while lower vitamin D levels are associated with allergic rhinitis, atopic dermatitis, and urticaria, supplementation shows beneficial effects mainly in these conditions, with no significant effect observed in asthma or food allergy, underscoring context-dependent immunomodulation. **(B)** Dynamic biomarkers of treatment response. In thyroid-associated ophthalmopathy, changes in vitamin D levels during therapy (Δ25(OH)D), rather than baseline levels, predict glucocorticoid response, highlighting vitamin D responsiveness as a clinically relevant trait. **(C)** Integrative multi-parameter models for risk stratification. In pediatric asthma, ferritin and eosinophil percentage are independent predictors of exacerbations, while vitamin D displays a threshold association with psychological stress-induced asthma. Integration of US NHANES data with untargeted metabolomics approaches identifies calcidiol as a potential biomarker associated with this phenotype. **(D)** Vascular protection and clinical gaps in Kawasaki disease. Vitamin D may contribute to vascular protection through NF-κB modulation, endothelial progenitor cell enhancement, and platelet regulation, although prospective trials are required to establish clinical benefits. Collectively, these advances illustrate the transition of vitamin D research from association to mechanism and ultimately toward personalized immune modulation. 25(OH)D, 25-hydroxyvitamin D; Δ25(OH)D, change in serum 25-hydroxyvitamin D concentration; ECs, endothelial cells; EPC, endothelial progenitor cell; MPV, mean platelet volume; NF-κB, Nuclear factor kappa-light-chain-enhancer of activated B cells; US NHANES, United States National Health and Nutrition Examination Survey; PSA, psychological stress-induced asthma; TAO, thyroid-associated ophthalmopathy.

Against this evolving scientific landscape, the present volume builds upon the conceptual framework established in the previous volumes of this Research Topic (15, 21), and reflects the continuing maturation of the field. Building upon the foundational knowledge of vitamin D’s effects on the transcriptome, gut microbiota, and systemic inflammation (Volume I) and its role in immune regulation across diverse conditions from allergies to cancer (Volume II), this third volume represents a significant maturation of the field. The focus has shifted from merely confirming associations to delving deeper into specific mechanistic pathways, identifying predictive biomarkers, and exploring the nuances of vitamin D’s action across different diseases and populations. The contributions presented in this volume span complementary areas of vitamin D immunomodulation, from disease-specific evidence in allergic disorders to clinical risk prediction in childhood asthma and the emerging role of vitamin D as a predictive biomarker and therapeutic target in other immune-mediated diseases, as summarized in **Figure 1**.

## 2 Key contributions from volume III

### 2.1 From broad association to disease-specific evidence: an umbrella review on allergic diseases

The umbrella review by Xu et al. provides a monumental synthesis of the evidence linking vitamin D to allergic diseases. By systematically analyzing 14 meta-analyses encompassing over 66,000 participants, this work establishes a clear hierarchy of evidence. The authors demonstrate that while lower serum vitamin D levels are consistently associated with conditions like allergic rhinitis (AR) and urticaria, the therapeutic efficacy of supplementation is disease-specific. Vitamin D supplementation proved beneficial for symptom improvement in AR, atopic dermatitis (AD), and urticaria, but showed no significant effect on asthma or food allergy. This critical finding highlights that the role of vitamin D in allergic disease is not uniform but is contingent upon the specific pathophysiology of each condition. It also reaffirms a key theme of our series: vitamin D acts as a context-dependent immunomodulator, with its benefits being most pronounced in specific disease settings.

### 2.2 Vitamin D, inflammation, and clinical outcome prediction: the cases of childhood asthma

Two original studies in this volume explore the intricate connections between vitamin D status, inflammation, and clinical outcomes in pediatric asthma, moving beyond simple correlations to more integrative models.

Wang et al. conducted an integrative analysis in children with asthma, investigating the interplay between vitamin D, ferritin, and eosinophilic inflammation in predicting acute exacerbations. Their study is a paradigm of the move toward multi-parameter risk stratification. They found that ferritin and eosinophil percentage were independent risk factors for exacerbation, whereas vitamin D showed an inverse association that was attenuated after multivariable adjustment. This suggests that while vitamin D deficiency may be a contributing factor, its effect is intertwined with other, more direct inflammatory drivers like iron metabolism dysregulation and type-2 inflammation. The study’s use of restricted cubic spline analysis and decision curve analysis showcases a sophisticated approach to assessing the clinical utility of these biomarkers.

Complementing this, Zhang et al. investigated the association between vitamin D levels and a specific asthma phenotype: psychological stress-induced asthma (PSA). Leveraging the large, population-based National Health and Nutrition Examination Survey (NHANES) database and validating their findings with an untargeted metabolomics study in a Chinese cohort, they identified calcidiol as a key metabolite associated with PSA. Their research reveals a nuanced, non-linear relationship, suggesting a threshold effect where the risk of PSA (depression) increases significantly when vitamin D levels fall below 67 nmol/L. This study is a prime example of how large-scale epidemiological data, combined with metabolomics, can uncover novel disease subtypes and their potential modifiable risk factors.

### 2.3 Vitamin D as a biomarker and therapeutic target in other immune-mediated conditions

The remaining articles in this volume broaden the scope of vitamin D research to other important clinical areas.

Wang et al. investigated the role of vitamin D in thyroid-associated ophthalmopathy (TAO), an autoimmune disease of the orbit. In a retrospective cohort of patients treated with intravenous glucocorticoids (IVGC), they made a pivotal discovery: the *change* in vitamin D levels during therapy (Δ25(OH)D), rather than the baseline level, was the strongest independent predictor of treatment response. This finding is conceptually important, suggesting that the body’s ability to replenish vitamin D stores may be more critical than the starting point. It also provides a tangible, actionable clinical biomarker, with an optimal cutoff of a +2.7 ng/mL increase associated with a better response.

The study by You et al. explores the association between vitamin D deficiency and the severity of comorbid adenoid hypertrophy (AH) and AR in children. In a cohort of 268 children, they found a strikingly high prevalence of deficiency (70.5% < 20 ng/mL) and a strong, dose-dependent association between lower 25(OH)D levels and more severe disease, as measured by A/N ratio, total IgE, and symptom scores. This research strengthens the link between vitamin D status and lymphoid tissue hyperplasia, providing a potential modifiable factor in managing this common pediatric comorbidity.

Finally, the comprehensive review by Yang and Zeng synthesizes the current evidence on Vitamin D and Kawasaki disease (KD). They present a compelling argument for the potential protective role of vitamin D, detailing the molecular mechanisms through which it could mitigate vascular injury. They explore how vitamin D may inhibit NF-κB activation to reduce endothelial inflammation, enhance endothelial progenitor cell function, and modulate platelet activity. Critically, they also identify major knowledge gaps, particularly the need for large, prospective trials to determine if adjunctive vitamin D supplementation can reduce the risk of intravenous immunoglobulin (IVIG) resistance and coronary artery lesions (CALs).

## 3 Concluding perspectives: towards precision immunomodulation

The articles in this third volume reflect the ongoing evolution of vitamin D research, from describing broad associations between vitamin D and the immune system toward a more precise and context-dependent understanding of its immunomodulatory functions. The overarching theme is the transition toward more individualized approaches to vitamin D-based immune modulation.

Several key insights emerge from this volume. Vitamin D should not be considered a universal therapeutic approach for allergic diseases, as its beneficial effects appear to be restricted to specific conditions, including AR, AD, and urticaria, while remaining unconfirmed for asthma and food allergy. Furthermore, dynamic changes in vitamin D status during treatment may provide more relevant clinical information than baseline measurements alone, highlighting the concept of individual vitamin D responsiveness. The immunomodulatory effects of vitamin D should also be interpreted within complex biological networks integrating metabolic, cellular, and clinical parameters, requiring sophisticated multi-parameter models. Finally, the combination of large-scale population analyses with high-resolution molecular approaches represents a promising strategy to validate epidemiological observations, identify novel biomarkers, and refine our understanding of the disease- and patient-specific effects of vitamin D on immune responses.

We anticipate that the insights presented here will contribute to further translational research and support the rational design of vitamin D-based interventions tailored to individual immune and metabolic profiles, with potential implications for patient care across immune-mediated diseases.
